# The Soft Occiput; a Contribution to the Physiology and Pathology of Early Childhood

**Published:** 1844-04

**Authors:** 


### Art. VI.

*Der weiche Hinterkopf; ein Beitrag zur Physiologie und Pathologie der ersten Kindheit.* Von Dr. C. L. Elsaesser.—*Stuttgart*, 1843. 8vo, pp. 230.

*The Soft Occiput; a Contribution to the Physiology and Pathology of Early Childhood.* By Dr. C. L. Elsaesser.—*Stuttgart*, 1843.

We have seldom met with a book for which its author can more fairly claim the merit of originality. The affection of which it treats, though it would appear to be neither unimportant nor infrequent, has not hitherto been noticed by medical writers. Dr. Elsässer's attention was first called to it accidentally five years ago; he has since studied the disease carefully, and the results of his investigations are embodied in the work before us. This work contains much that is both new and valuable, though we cannot help thinking that the author has occasionally ridden his hobby rather too hard. Be that as it may, however, we feel that we have not yet had opportunity sufficient for thoroughly testing the accuracy of all his statements, and shall therefore content ourselves with laying a simple abstract of them before our readers.

The disease described by Dr. Elsässer consists in a softening of the cranial bones, and consequent thinning of those parts of the skull, such as the occiput, which are much exposed to pressure. Hence its names of "the soft occiput," from its most striking symptom, or of *craniotabes*, expressive of the general disease of the cranial bones. Dr. Elsässer believes the affection to be a variety of rickets; that form namely which rickets assumes in the infant, while in after years it involves chiefly the bones of the trunk. So far too is it from being a rare disease, that in five years forty cases have come under the author's notice, and the consequences resulting from it are so serious that fourteen of these forty patients died.

Before entering on a description of this morbid state of the infantile skull, Dr. Elsässer makes some very interesting observations on the structure of the cranium in early life, and on its mode of growth, and points out the objects that are attained by its peculiarities in both these respects.

The new-born man occupies a lower position than the young of other mammals, with reference to the development and activity of his animal functions. He is unable to support his head, but lies in a horizontal posture, his head almost always resting on the occiput. Associated with this low state of activity of the animal functions is the comparatively undeveloped condition of the brain, and, as a necessary sequence, the imperfect ossification of its bony case. It is true indeed that the brain in the human fœtus bears a greater proportion to the weight of the body, than it does in the fœtus of other animals; but if the size of the human brain at birth be compared with that which it attains during the first few years of life, we cannot but regard its development as very incomplete. No organ except the spleen increases in size so rapidly during early life; for in the second year the weight of the brain is nearly double that which it was at birth; while according to Sömmering and the Wenzels, its growth ceases altogether in the seventh year. Hence it was necessary that the cranium should admit of the free growth of the brain; and accordingly we find this great end answered by the peculiar form of the fœtal skull, by its fontanelles and its unossified sutures.

In animals who are accustomed from birth to bear the weight of their head, and in whom it is consequently exposed to blows and concussions, any such softness of the bony capsule as exists in the human subject, would have been inconvenient and even dangerous; therefore in them the sutures are firmly closed, and the fontanelles ossified. In some of them, however, a provision exists for the growth of the skull in the overlapping of the temporal, occipital, and parietal bones where they are in contact with each other; thus, in the young of the pig, nearly a third of the concha of the occipital bone overlaps the parietal bones. This arrangement is usually found in the human subject only at that part where the squamous portion of the temporal overlaps the parietal bone; though in a few instances a slight overlapping of the sutures is likewise present. In all cases, however, the unossified fontanelles and unclosed sutures serve the double purpose of permitting the mechanical expansion of the skull by the enlargement of the brain, and of rendering the growth of the cranial bones themselves possible. Among these provisions for the growth of the brain and enlargement of the skull a peculiar importance is to be attached to the great fontanelle, which, according to Dr. Elsässer, progressively increases in size during the first nine months of infantile life. He examined its condition in seventy-five children; fifty of whom were under fourteen months old, and in all of them it was still open; twenty-four were between fifteen months and two years, and in ten of them it was still open, and in fourteen it was closed. In the only child whose age exceeded two years, the anterior fontanelle was likewise closed.

"The dimensions of the anterior fontanelle at successive periods of three months were as follows:

| Trimensual periods. | Number of children. | Average diameter of the Fontanelle. |
|---|---|---|
| From 1st to 3d month | 10 | 9·60 lines. |
| 4th — 6th | 15 | 11·93 |
| 7th — 9th | 7 | 13·90 |
| 10th — 12th | 13 | 11·88 |
| 1st — 12th During which the Fontanelle is always open. | 45 | 11·60 |
| From 13th to 15th month | 9 | 7·78 |

"Of these 9 children the fontanelle was closed in 3; in 1 it was five lines, in the others from ten to fifteen lines in diameter.

From 16th to 18th month ... 8

"Of these 8 children the fontanelle was closed in 4; in the rest it was two, three, nine, and ten lines in diameter.

From 19th to 21st month ... 5

"In 2 it was closed; in the others five, twelve, and twelve lines in diameter.

From 22d to 24th month ... 7

"In 5 it was closed; in the others nine and fifteen lines in diameter." (p. 10.)

"On a comparison of the size of the anterior fontanelle in children of about the same age—that is to say, belonging to the same *trimester*—a greater discrepancy is observed the older that the children are. Thus it varied—

| | | | | |
|---|---|---|---|---|
| "In the 1st Trimester from | 8 | to | 12 lines. | |
| 2d | 6 | | 16 | |
| 3d | 5 | | 17 | |
| 4th | 5 | | 19 | |
| 5th | 0 | | 15 | etc." (p. 12.) |

If nature, then, do so carefully maintain a membranous interspace between the bones of the skull, we may fairly assume that some good end is thereby answered. The continuance of a longitudinal fissure, such as the sagittal suture, would not have answered this end; for it would have been liable to be diminished by pressure, while it would also have been gradually obliterated by the growth of the bones. From the very form of the fontanelle, however, this result, as Dr. Elsässer explains at pp. 13-14, does not ensue, while its approximation to a circular shape has the advantage of affording a large space in a small room. The uses of the anterior fontanelle are not merely of a negative kind, but it serves as a sort of safety-valve, bulging outwards when the brain is congested, sinking inwards when its circulating fluid is diminished; thus preventing the serious results either of congestion or anemia.

With reference to the varying size of the anterior fontanelle in children of the same age, it is fair to conclude that an unusually small fontanelle depends on the great activity of the ossification of the cranial bones; and a remarkably large fontanelle on its preternatural slowness, a condition usually associated with congenital weakness of the constitution. With this unusually wide fontanelle a preternaturally large head is generally combined, for the tardy ossification of the bones favors their superficial growth.

But, although the anterior fontanelle is yielding, yet the bones of the skull in early infancy are in general firm and resistent, and cannot be indented by moderate pressure, except slightly in the immediate neighbourhood of the anterior fontanelle. Dr. Elsässer found, however, that in seventeen of the seventy-five children whose heads he examined, the cranial bones were in some parts pliable, and admitted of being depressed by the finger. Not one of these children, however, was under three months old; a fact which sufficiently proves that this condition cannot be regarded as congenital. These yielding parts of bone, too, were all, without exception, at the occiput; and though the children in whom they existed were tolerably healthy, yet Dr. Elsässer infers that this condition was an incipient stage of craniotabes.

The author next calls attention to the changes which the interior of the skull undergoes in the course of years. From being comparatively smooth, it comes to present a series of depressions, worn, as he believes, by the continued pressure of the brain. In support of this theory of their origin, he adduces the fact that these indentations are found especially in the occipital bone, while it is that very part which bears the chief pressure of the brain; the child lying almost constantly in the horizontal posture for many months, and afterwards spending a great part of its time in that position.

The second chapter treats of the morbid anatomy of this affection. The skull of children affected with craniotabes is soft, easily cut, its bones have lost their compact structure, have become softer, more vascular, more pliable, and similar to the spongy bones. They have lost their smooth surface and fibrous texture, and have become rough and porous; a state produced by the diminution of their earthy constituents, and a disintegration of their tissue. The canaliculi of the bones become dilated, more cellular, and communicate freely with other canaliculi; changes precisely similar to those which Miescher describes as being produced by rhachitis in other bones. Besides this condition of the cranial bones in general, various spots will be found at the posterior part of the skull, where the osseous substance is extremely attenuated, or even totally wanting, and these spots will be found to correspond to some of the gyri of the brain. These attenuated or perforated spots are found mostly in the occipital or parietal bones, and in the immediate neighbourhood of the lambdoid suture. The bones in that situation are yielding and elastic, like stiff paper or tinsel, and, on laying the child with its head upon a hard surface, they will be depressed some lines. When there is actual loss of bony substance, these holes can be felt distinctly in the midst of the stiff and elastic cranial wall. Occasionally these holes are very numerous; Dr. Elsässer once counted thirty in a single skull.

Minute details of the history of thirty-seven cases follow, and on these Dr. Elsässer grounds his description of the general features of the disease. The children in whom it exists are mostly of a weakly constitution, and distinguished by a disposition to tardy development. They may, notwithstanding, have tolerably good health, and in their second or third year may grow rapidly, and become as robust as other children. They are further characterized by a general softness of their bony structures, they are usually restless, unquiet, often moaning, subject to disturbance of their digestive organs, and more liable than others to convulsive attacks. Sometimes, however, it comes on in children previously strong, healthy, and well nourished, and an unusual development of fat has seemed, in some instances, rather to predispose to its occurrence. In either case, some accidental cause generally gives rise to the disease; it may follow a blow, or may succeed to an attack of catarrh, or to disturbance of the digestive organs. The symptoms which accompany it arise from two sources,—the general dyscrasia, and the local lesion, but the line between the two cannot be drawn with any great accuracy.

The symptoms of craniotabes, for the most part, make their appearance about the third or fourth month; when the children begin to sleep ill, or their former restlessness is notably increased. They sleep with their eyes half open, roll their head about much, and bore with it in the pillow. At the same time, too, they perspire much, and this perspiration is especially profuse about the head. During the day-time they are surprisingly cheerful, and seem as though nothing ailed them, unless they fall asleep, when the state of disquiet again returns, and they become extremely impatient and fretful if laid with the head upon a hard pillow. Sometimes the head appears tender, so that the occiput cannot be touched without pain, while the children are fond of lying on the belly, and will often rub their face against the pillow. The whole nervous system seems highly excitable, and the slightest cause suffices to alarm the child.

The growth of hair on the head of children thus affected is naturally scanty, and after the disease has existed for some time the occiput becomes nearly bald, from being incessantly rubbed against the pillow. There is more or less disturbance of the digestive functions, and catarrh and diarrhea are frequent, though not severe. Sometimes, though not always, rhachitic deformity of the chest supervenes, and the patient then suffers from frequent cough. None of these phenomena, however, impart to the disease its grave, and often fatal character. This depends on the convulsive seizures, which occurred in fourteen out of twenty-nine cases, and, in ten of these, proved fatal. These attacks were either simple convulsions, or else of a tetanic character, and, in some instances, both of these were combined. They came on once, or oftener, in the course of the day, but sometimes an interval of days, or even weeks, would elapse between their recurrence. In the intervals the child was comparatively healthy; that is to say, that no other than the general symptoms of craniotabes were present. Frequently death took place after several returns of the attacks, and, when this was the case, there was seldom a complete intermission between the seizures, but a febrile condition, with symptoms of persistent cerebral irritation, continued for some days before the fatal event.

"In those fatal cases, in which post-mortem examinations were made, a marked state of congestion, or inflammation of the membranes of the brain, or spinal cord, was always discovered.

"These anatomical alterations of the nervous centres plainly indicated the cause of the attacks, and led likewise to the inference, from analogy, that, in the earlier stages of the disease, when intervals of health occurred between the attacks, though no permanent condition of congestion or inflammation existed, still a temporary congestion of the brain must have occurred, and given rise to the convulsions."

In those cases in which the convulsions partook of a tetanic character, either the breath was suspended, owing to the spasm extending to the respiratory apparatus, or respiration went on with more or less obvious disturbance. This affection of the respiratory movements occurred in four children, and in them, as well as in those instances where the convulsions presented a simply tetanic character, the attacks came on suddenly, and without any preliminary symptoms.

" ........The muscles of the back, of the eyes, face, and limbs, became firmly contracted, and, at the same time, respiration was suspended. The whole body became cold as a corpse, the face grew livid, and was covered with a cold sweat. Sometimes, before the power of respiration returned, general relaxation of the muscles took place, so that the child's head and arms sank powerless, and conveyed to bystanders still more forcibly the impression that life had fled. In some instances, indeed, paralytic relaxation of the muscles seemed to coexist with the suspended respiration from the very beginning of the attack, or at least the previous contraction of the muscles was of such brief duration as to be scarcely observable. Occasionally, a sudden screech announced the commencement of the attack; hasty, laborious expirations, with proportionably long inspirations, betokened its close. When respiration was once more established, the child would sink down exhausted, and usually fell asleep." (p. 137.)

The age at which craniotabes usually commences, its modes of termination, its dangers, and its influence on infantile mortality, are next investigated. Dr. Elsässer concludes that the disease may commence in the first three months of infantile life, and perhaps soon after birth; but that it usually comes on in the course of the second three months of infancy. It should be observed, however, that he had the opportunity of determining the date of its commencement only in three instances, in which he saw the children before the disease began, and, at a subsequent period, found them presenting marks of its existence. In those instances in which recovery takes place, the skull usually regains its natural firmness within the first year of life, or at least between the eighth and thirteenth month. Recovery is announced by the gradual remission of the symptoms, and return of the natural condition of the skull.

With reference to the mortality produced by this disease, it appears that of twenty-nine children affected by it, fifteen recovered, fourteen died; of whom ten died in convulsions. In round numbers, then, it may be stated, that craniotabes proves fatal to half of those affected by it; a result which sufficiently proves the disease to be of serious importance, as well as of frequent occurrence.

The essential nature of the disease is the subject of the next chapter. Dr. Elsässer calls attention to the fact, that no condition of the cranial bones, such as he here describes, exists at any period of fœtal life, or is found as a result of simple arrest of development. Some writers, indeed, who appear to have met with cases of this affection, attribute them to that cause; but at no period of development do we find patches of membrane surrounded by bone, though the sutures be never so widely open; nor should it be forgotten that the parietal bone in which several of these membranous spots are often found is formed from a single point of ossification. The attenuation of the skull is the simple result of pressure on the softened bone, as is shown by the holes existing only about the occiput, and corresponding exactly to the convolutions of the brain. That portion of the skull, too, is always affected on which the head rests, and which, consequently, is subjected to pressure from the brain within, and the pillow on which the infant reposes without. Not only the mere weight of the brain and its rapid growth concur to produce the attenuation of the skull, but its rhythmical movements also contribute to the same effect. These, then, are its proximate causes; its remote cause is to be found in the relatively small degree of development of the animal functions and of their organ, the brain, in the young of the human subject.

What, however, occasions that morbid softness of the bones of the skull, which allows of their being thus attenuated by pressure? The author, as we have already mentioned, considers it to be of rhachitic origin; in many instances, indeed, signs of rickets existed in different parts of the body. Writers, indeed, have usually regarded infantile life as free from the attacks of rickets; and have dated its commencement from the first, second, or third year of childhood. But Dr. Elsässar observes:

"According to the result of our observations, infancy enjoys no immunity from this disease; it is indeed of by no means rare occurrence, and, at any rate, is much more frequently met with than was commonly supposed. It manifests itself in infancy chiefly in softening of the cranial bones, and consequent thinning of the occiput. Craniotabes is the form which rhachitis assumes in infancy.

"This form of rickets having hitherto been overlooked, it necessarily followed that the real commencement of the rhachitic *dyscrasia* was not apprehended. It was erroneously supposed that the disease did not begin until the end of the first year of life, or even later; its commencement and its course were described only as they affected the rest of the skeleton, while the skull was either unnoticed, or was assumed to possess a kind of immunity from the disease.

"According to our observations, however, rhachitis is often almost entirely limited to the bones of the skull They may, too, recover from this diseased condition without any deformity of the rest of the skeleton having ever been produced, and this whole course of rickets may be run within the first year of life.

"Various attempts have been made to determine the chronological order in which the successive effects of rickets on the skeleton are produced. The fact is unquestionable, that some parts of the skeleton may present a high degree of morbid softening and deformity, while other parts are not affected in any appreciable measure. This is especially true of rickets affecting the skull.

"Another fact is, that skeletons softened by rickets may have regained in some parts their natural firmness, while other parts are still soft and deformed, or are just becoming so. Thus, for instance, it is not unusual for craniotabes to have disappeared when the bones of the thorax, spine, or extremities are beginning to be curved and morbidly enlarged. Any one who, in such a case, is ignorant of the existence of the former affection would date the commencement of the whole disease from the appearance of the latter symptoms.

"The erratic character of rickets is a fact beyond dispute. The rule according to which this wandering of the disease takes place is intimately connected with the phases of development of the principal parts of the body.

"It usually establishes itself in whichever part of the body the vegetative and functional endowments are in the most active and energetic state of development. In this respect, the head, thorax, and extremities may be distinguished. With the changes in the development of the viscera, there takes place a corresponding change in the development of the bones which inclose them." (pp. 152-3.)

We cannot follow Dr. Elsässer through the various illustrations by which he supports this theory, and shows how the head, chest, and locomotive organs become, in succession, the seat of rickets. He regards the convulsions which were present in nearly half the cases as a secondary occurrence, induced by the irritation and excitement of the imperfectly shielded brain. That condition of the skull which leaves the brain thus incompletely defended, he believes himself justified in regarding as a frequent—perhaps the most frequent—cause of convulsions in infancy, and especially of those in which the attacks are interrupted by intervals in which the patient is free from them. From these observations he passes to a very lengthened comment on laryngismus stridulus, the drift of which is to establish that the affection depends on primary disorder of the brain rather than of the respiratory nerves; and that this cerebral disorder results, in the greater number of cases, from craniotabes. In support of this opinion he appeals to cases of laryngismus recorded by different writers, in which either rickets or some morbid condition of the cranial bones is stated to have existed.

The predisposing cause of this disease is to be sought in a congenital weakness of the constitution, with which is usually associated a tardy development of the whole body, and especially of the osseous system. The disease often runs in families, and very fat children seem peculiarly liable to it; and in some in whom it occurred this abundance of fat was not associated with laxity of fibre or any indication of other than robust health. It is often developed after some severe acute or chronic disease, especially catarrh or other affections of the respiratory organs; next to which in frequency derangements of the bowels seem to give rise to its occurrence. Impure air, damp, and want of cleanliness are also powerful exciting causes. The disease, too, is more frequent in winter than in summer—a difference which Dr. Elsässer attributes not to the cold of that season, but to the fact that the rooms of the poor are then more strictly closed than in summer, by which means pure air is almost entirely excluded, while the stoves are so heated as constantly to maintain an unnaturally high temperature.

The treatment of the disease may he summed up briefly: Judicious dietetic management is of quite as much importance as therapeutical measures; but the rules by which it is to be regulated do not differ from those that would apply to delicate children in general, except in one particular. This point regards the pillow for the head, which should be soft, elastic, cool, and so formed as not to allow the head to sink too deep into it. A horse-hair pillow combines these advantages, but they are still more certainly attained by cutting in it a pear-shaped hole, with its apex directed downwards, and a diameter of two and half to three inches at its broadest part, in which the occiput is received without the soft bone resting upon any surface beneath.

The medicinal treatment of the disease consists in the administration of preparations of iron, which appeared to answer better than the oleum jecoris aselli, or than quinine or other tonics. The carbonate was the form in which it was most frequently used by Dr. Elsässer. The constipation which was prone to occur was best removed by small doses of aloes, besides which the regular use of *Eichelkaffee*,* in children who were no longer at the breast, seemed often to obviate that occurrence. With these internal remedies the employment of tonic baths was combined. Baths containing decoctions of aromatic herbs or an admixture of iron, were used with benefit; but none appeared to do so much good as tan-baths. These were prepared by boiling two or three handfuls of ground oak-bark, such as is used by tanners, in two or three quarts of water, for half an hour, and then adding the decoction to the bath. These baths should not be too warm, and their temperature should be gradually reduced till they are as cool as the child can bear without shivering. Cold sponging may be advantageously combined with the use of the bath.

The treatment of the different complications does not present anything of importance, beyond the fact that the above-mentioned plan is usually to be persevered in in spite of them. The convulsions are to be treated by leeches, mercurials, and counter-irritants.

We have now presented our readers with an outline of this interesting monograph. We have no liking for hasty criticism, and therefore offer no comment on the writer's statements. They seem, however, to be made in all good faith, and as the result of careful investigation; and we trust that the subject will engage the attention of others who are placed in a position for observing the diseases of children.

* The *Eichelkaffee,* or acorn coffee, which is a favorite remedy in Germany for various cachectic affections of children, is prepared by mixing half a tablespoonful or more of roasted acorns in powder with half that quantity of coffee, and preparing with it a beverage which may be taken twice a day, and when flavoured with milk and sugar, like ordinary coffee, has not by any means a disagreeable taste.

